# The Role of Cannabinoids in Bone Metabolism: A New Perspective for Bone Disorders

**DOI:** 10.3390/ijms222212374

**Published:** 2021-11-16

**Authors:** Federica Saponaro, Rebecca Ferrisi, Francesca Gado, Beatrice Polini, Alessandro Saba, Clementina Manera, Grazia Chiellini

**Affiliations:** 1 Department of Pathology, University of Pisa, 56100 Pisa, Italy; federica.saponaro@unipi.it (F.S.); francesca.gado@for.unipi.it (F.G.); beatrice.polini@farm.unipi.it (B.P.); alessandro.saba@unipi.it (A.S.); 2 Department of Pharmacy, University of Pisa, 56100 Pisa, Italy; rebecca.ferrisi@phd.unipi.it

**Keywords:** endocannabinoids system (ECS), bone remodeling, cannabinoid receptor type 2 (CB2R), osteoporosis, bone cancer

## Abstract

Novel interest has arisen in recent years regarding bone, which is a very complex and dynamic tissue deputed to several functions ranging from mechanical and protective support to hematopoiesis and calcium homeostasis maintenance. In order to address these tasks, a very refined, continuous remodeling process needs to occur involving the coordinated action of different types of bone cells: osteoblasts (OBs), which have the capacity to produce newly formed bone, and osteoclasts (OCs), which can remove old bone. Bone remodeling is a highly regulated process that requires many hormones and messenger molecules, both at the systemic and the local level. The whole picture is still not fully understood, and the role of novel actors, such as the components of the endocannabinoids system (ECS), including endogenous cannabinoid ligands (ECs), cannabinoid receptors (CBRs), and the enzymes responsible for endogenous ligand synthesis and breakdown, is extremely intriguing. This article reviews the connection between the ECS and skeletal health, supporting the potential use of cannabinoid receptor ligands for the treatment of bone diseases associated with accelerated osteoclastic bone resorption, including osteoporosis and bone metastasis.

## 1. Introduction

**Figure 1:**
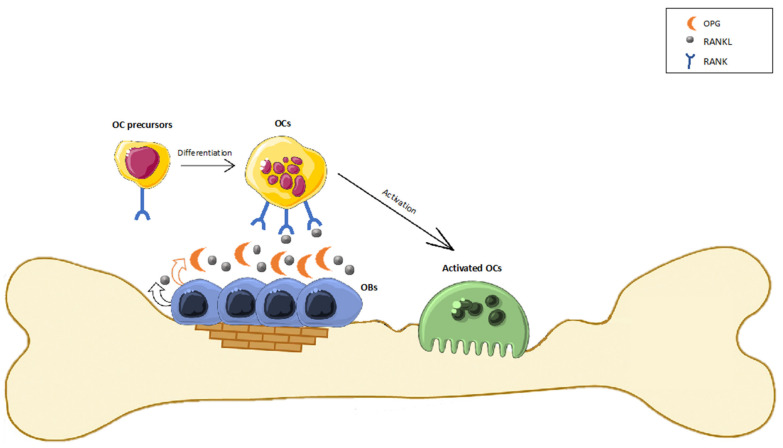
Bone remodeling process. OBs express RANK-L which promotes osteoclastogenesis following its binding with specific receptor RANK onto the OCs precursors’ surface. This binding induces the maturation of preosteoclasts into mature OCs, resulting in resorption of bone tissue and the release of growth factors. OBs also produce osteoprotegerin (OPG), which acts as a decoy receptor for RANK-L, inhibiting OC formation by blocking RANK-L binding to RANK and stimulating OCs to induce apoptosis. The OPG/RANK-L ratio is a better indicator of bone remodeling status: a high ratio represents bone formation, while a low ratio favors bone resorption.

The skeleton is an extremely specialized and dynamic organ that undergoes continuous regeneration. The maintenance of physiological bone remodeling and systemic mineral homeostasis requires a fine balance between bone formation and bone resorption. This process involves the concerted actions of two types of bone cells: osteoblasts (OBs), which belong to stromal cells and have the capacity to produce new bone, and osteoclasts (OCs), which are derived from monocytes and remove old bone [1]. The bone remodeling process is a cycle consisting of an initial resorption phase, driven by the multinucleate OCs digesting mineralized tissue, a reversal phase with mononuclear cells on the bone surface, and a bone formation phase with the OBs that lay down a new mineralized matrix. The resorption and formation phases are tightly coupled thanks to the receptor activator of the nuclear factor-κB (RANK)/RANK Ligand (RANKL)/osteoprotegerin (OPG) axis [2] (Figure 1). The bone remodeling process occurs simultaneously in different foci in the skeleton, and it is crucial to save bone from damages, to respond to mechanical and loading stimuli, and to control calcium homeostasis [3,4]. In healthy bone, RANKL, which is primarily expressed in OBs, promotes osteoclastogenesis following its binding with the specific receptor RANK onto the OCs precursors’ surface. This binding induces the maturation of preosteoclasts into mature OCs, resulting in the resorption of bone tissue and the release of growth factors, such as the transforming growth factor-β1 (TGF-β1), which in turn stimulate the OB formation. A key preliminary step in downstream signaling after RANKL ligation to RANK consists of the binding of TNF receptor-associated factors (TRAFs). Among TRAFs, TRAF6 has an essential function in the OCs and leads to the activation of mitogen-activated protein kinases (MAPKs) and the transcription factors nuclear factor-κB (NF-κB) and activator protein-1 (AP-1). The subsequent signaling process is characterized by the amplification of the c-Fos expression, which interacts with the nuclear factor of activated T-cells cytoplasmic 1 (NFATc1) to trigger the transcription of the osteoclastogenic gene [5].

RANK/TRAF-mediated protein kinase signaling activates at least seven signaling pathways: four directly mediate OC formation (inhibitor of NF-κB kinase (IKK)/NF-κB, c-Jun N-terminal kinase (JNK)/activator protein-1 (AP-1), c-myc, and calcineurin/NFATc1), and three mediate OC activation (Src and MKK6/p38/MITF) and survival (Src and extracellular signal-regulated kinase) [6,7]. OBs also produce OPG, which acts as a decoy receptor for RANKL, inhibiting OC formation by blocking RANKL binding to RANK and stimulating OCs to induce apoptosis.

Bone remodeling is a tightly controlled process, which involves both local and systemic factors, including growth factors and hormonal regulators, with Calcitonin (CT), parathyroid hormone (PTH), 1,25(OH)_2_ vitaminD_3_, and sex hormones being the major hormonal regulators of osteoclastic bone resorption [8,9]. In particular, lipopolysaccharide (LPS), Vitamin D3, and pro-inflammatory cytokines can increase OC formation via up-regulating the expression of RANKL and/or down-regulating OPG in the OBs; in contrast, the OPG expression is decreased by prostaglandin E2 (PGE2), PTH, glucocorticoids, and the insulin-like growth factor-1 (IGF-1) [10]. RANKL and RANK are under the tight control of the female sex hormones estradiol and progesterone. Estradiol promotes bone formation by increasing OPG expression. When circulating estradiol levels drop during menopause, RANKL-stimulated osteoclast activity increases, leading to a progressive decrease in bone mass [11]. Undeniably, an imbalance of the RANK/RANKL/OPG signaling pathway leads to pathological processes, including postmenopausal osteoporosis, hypogonadism, androgen deprivation therapy (ADT)-induced bone loss, and rheumatoid arthritis. In addition, the triad RANK/RANKL/OPG plays a key role in oncological phenomena, modulating cancer cells migration and angiogenesis and thus controlling the development of bone metastases.

There is accumulating evidence to suggest that the endocannabinoid system (ECS) is involved in the regulation of bone cell activity and bone remodeling and thus plays an important role in the regulation of bone disease, including cancer-induced bone disease (CIBD) [12,13,14]. This review summarizes in vitro and in vivo findings on the action of the cannabinoid receptor ligands in skeleton health and pathology, associated with recent advances in the development of highly sensitive analytical methods for the determination and quantization of endogenous cannabinoid ligands (ECs) in plasma and tissue samples.

## 2. An Overview of the Endocannabinoids System (ECS)

The ECS is a complex lipid signaling system recognized for playing an important role in all aspects of mammalian physiology and pathology, ultimately contributing to the homeostasis of the organism, which encompasses the brain, endocrine, and immune system, to mention a few [15]. It comprises cannabinoid receptors (CBRs), endocannabinoid ligands (ECs), and the enzymatic machinery that drives the biosynthesis, degradation, and transport of ECs [16,17]. CBRs belong to an extensive family of class-A G-protein coupled receptors (GPCR) and include two principal subtypes: cannabinoid receptor type 1 (CB1R), and cannabinoid receptor type 2 (CB2R), which were isolated and cloned only during the 1990s [18]. Moreover, the existence of additional CB-like receptors is well-documented. Among them, the transient receptor potential vanilloid type-1 (TRPV1) and the orphan GPCRs, GPR55, and GPR18 are all activated by cannabinoids and therefore considered atypical CBRs [19].

The CB1R has a ubiquitous distribution. It is predominantly expressed in all brain structures as the “brain cannabinoid receptor”, but it is also found in peripheral organs and tissues, including the gastrointestinal tract and the cardiovascular and reproductive systems, as well as the skeletal muscle, bone, and adipose tissue [20]. CB1R has been implicated in various disorders. However, the direct modulation of CB1R by agonists or antagonists has been associated with adverse psychiatric effects, such as depression, anxiety and suicidal ideation, thus limiting the clinical development of such agents [21].

Besides the canonical long-form, recent research has demonstrated alternative splicing of the CB1R gene, resulting in two isoforms, CB1aR and CB1bR, which differ in their N-terminus sequence, affecting their pharmacologic properties [22]. Characterization of the expression patterns of these isoforms has revealed that the full-length CB1R represents the dominant isoform in the brain and is responsible for the behavioral and psychotropic effects evoked by cannabinoids; the isoform CB1aR is predominantly expressed in the central nervous system, although its levels are negligible compared to the central CB1R, and the isoform CB1bR is highly expressed in pancreatic β-islet cells and hepatocytes and is involved in regulating metabolism [23,24].

The CB2R was first detected in peripheral cells and tissues belonging to the immune system, but over time, its expression has also been clearly documented in the central nervous system (CNS), e.g., in the microglial cells, astrocytes, and some subpopulations of neurons, particularly under neuroinflammatory conditions [25].

Although many features of the CB2R gene structure, regulation, and variation remain poorly characterized in comparison to the CB1R, some studies have led to the identification of two different human CB2R isoforms (CB2aR and CB2bR). While CB2aR expression was predominantly observed in the testis and in the brain, suggesting a major involvement in neuroprotection, isoform CB2bR was mainly detected in the spleen and leukocytes. Similarly to CB1R, the identification of CB2R isoforms should be taken into account while developing CB2R-based therapeutic agents [22,26].

CB1R and CB2R signal through G_i/o_ proteins and are thereby negatively coupled to adenylyl cyclase (AC), leading to a decrease in intracellular cAMP levels and the subsequent inhibition of protein kinase A (PKA). In addition, they stimulate mitogen-activated protein kinases (MAPK) signaling pathways, including extracellular signal-regulated kinase 1/2 (ERK1/2), c-Jun N-terminal kinase (JNK), and p38. CBRs are able to modulate cell growth and death not only via MAPK signaling but also via the phosphoinositide 3-kinase (PI3K)/protein kinase B (AKT) pathway [22,27]. Moreover, CBRs inhibit certain voltage-sensitive calcium channels, stimulate inwardly rectifying potassium channels (GIRKs), and recruit β-arrestins, among other actions [28].

**Figure 2:**
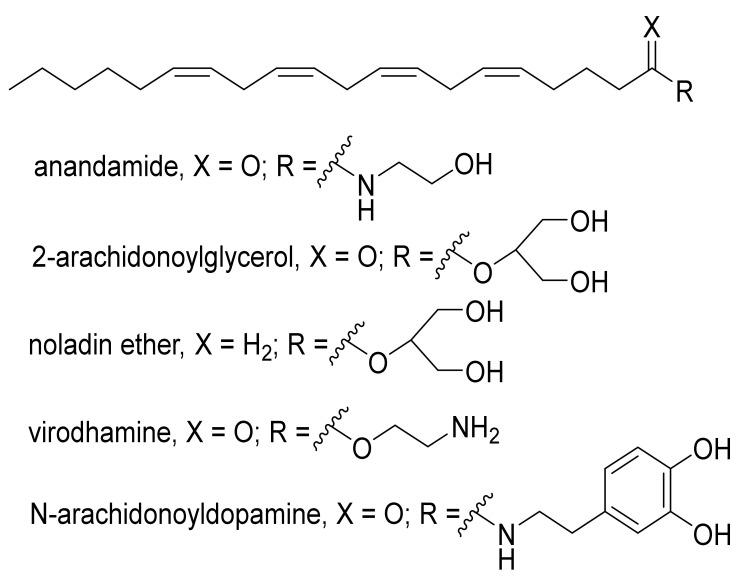
Structures of the main endocannabinoids (ECs).

CBRs are activated by ECs, a class of endogenous lipid messengers which are synthesized from the phospholipids of the cell membrane. The main ECs are *N*-arachidonoylethanolamine (anandamide, AEA) and 2-arachidonoylglycerol (2-AG) (Figure 2). Although both contain arachidonic acid in their structures, their routes of synthesis and degradation in vivo are almost completely distinct and are mediated by different enzymes. Briefly, AEA is synthetized by the *N*-acyl phosphatidylethanolamine phospholipase D (NAPE-PLD) enzyme from *N*-arachidonoyl phosphatidylethanolamine, and then, it is degraded by membrane-associated fatty-acid amide hydrolase (FAAH). 2-AG is produced from 2-arachidonoyl-containing phospholipids, primarily arachidonoyl-containing phosphatidyl inositol bis-phosphate (PIP2), under the action of phospholipase C (PLC) and the diacylglycerol lipases alpha and beta (DAGLα and DAGLβ) and hydrolyzed by monoacylglycerol lipase (MAGL) [29]. These ligands have complementary as well as divergent features which lead them to have distinct physiological roles. 2-AG functions as a full agonist of both CBRs, while AEA is a partial agonist for CB1R and CB2R [30]. It is important to notice that 2-AG, in addition to serving as an endogenous ligand for the CBRs, is an important source of arachidonic acid used for prostaglandin synthesis in the brain, liver, and lung but not in the gut, heart, kidney, or spleen. Thus, the management of 2-AG synthesis and degradation can have effects that are independent of the ECS [31]. Other lesser-known ECs also interact with the CBRs, including virodhamine, *N*-arachidonoyldopamine (NADA), and noladin ether (Figure 2).

## 3. Detection of ECs in Biological Fluids, Hair, and Tissues

Over the years, many analytical methods for the quantification of the main physiologically occurring cannabinoids, namely AEA and 2-AG, in different biological samples, such as plasma, serum, tissues, saliva, cerebrospinal fluid, and hair from humans, rats, and mice, have been reported in the literature, providing important insights into the physiology and pathology of the ECS and offering new therapeutic perspectives. These analytical methods are essentially based on high-performance liquid chromatography with UV (HPLC-UV) or fluorescence (HPLC-FL) detection, HPLC or ultra-high performance liquid chromatography (UHPLC) coupled with mass spectrometry, or GC coupled with mass spectrometry (GC-MS) [32,33,34,35,36,37]. Among these methods, tandem mass spectrometry, coupled to HPLC or UHPLC-MS with stable isotope dilution, is mostly used as the lack of chromophoric or fluorescent functional groups, as well as the very low concentrations in most biological samples, makes necessary the chemical derivatization of the molecules of interest prior to the analysis, either in the HPLC-UV or the HPLC-FL analytical method. Although not strictly necessary, a proper derivatization could also offer increased volatility and sensitivity in GC–MS. Nevertheless, the quantification of ECs is typically carried out by tandem mass spectrometry coupled to HPLC or UHPLC-MS with stable isotope dilution, which, as it does not need complicated sample preparations, offers high sensitivity and selectivity [35–37]. However, pretreatment of the sample may still be necessary in order to efficiently remove interfering compounds causing ion suppression or enhancement, which in electrospray ionization (ESI) and atmospheric pressure chemical ionization (APCI) cannot be avoided, possibly leading to chemical and physical sample degradations. In addition, 2-AG spontaneously isomerizes to 1-AG (1-arachidonoylglycerol) through an acyl group migration, and this process is augmented under elevated temperatures and high pH values. On the other hand, toluene used as a solvent for liquid-liquid extraction (LLE) minimizes the matrix-effect, thus reducing 2-AG isomerization and degradation, keeping a high extraction yield in the analysis of human plasma and urine samples [38,39,40]. In contrast, AEA is quite stable under common experimental conditions (i.e., methanol/chloroform extraction solvents) [39]. Sample preparation largely depends on the nature of the biological sample. However, the above-mentioned LLE and solid-phase extraction (SPE) are probably the most used techniques for lipid removal from the matrix [27,39,41,42,43,44]. The usual LLE protocols make use of chloroform/methanol, mixed in several ratios, to isolate all classes of lipids in the samples, including the phospholipids that contribute to the matrix-effects. Mixtures of ethyl acetate/hexane, acetate/heptanes, or acetone/toluene have been used too, as well as pure toluene, which offers the advantage of a minimal isomerization of 2-AG to 1-AG [32]. Many SPE materials are also available for the typical off-line sample preparation; among them, lipophilic reverse- and mixed-phase materials, such as octadecyl silica (C18) or copolymer- and normal-phase materials such as silica, provide good results [27]. This technique is particularly convenient when a full automation through on-line coupling with LC–MS/MS devices is possible as it improves the method throughput. Commercial devices from different producers, or custom-assembled devices, can be used for this purpose. The latter include devices outfitted with an on-line cleanup/enrichment section such as that used by Fanelli et al. [34], which was strictly derived from the high throughput SPE-HPLC-MS/MS equipment utilized by Koal et al. for the quantification of immunosuppressants in whole blood samples [35] and by Saba et al. for the quantification of cortisol and of some of its metabolites in urine [36]. The cleanup/enrichment stage made use of an Applied Biosystems (Waltham, MA, USA) POROS perfusion column as an SPE cartridge, which worked on the basic principle of the well-known high-turbulence liquid chromatography online extraction developed by Cohesive Technologies [37]. However, the on-line purification process was preceded by LLE with toluene, which improved the analyte extraction, recovery, and selectivity and minimized the 2-AG isomerization with respect to the different extraction solvents. Besides AEA, 2-AG, and its isomerization product, 1-AG, Fanelli et al. also monitored some of the bioactive lipids in human plasma, namely *N*-acylethanolamines (NAEs), such as *N*-palmitoylethanolamine (PEA) and *N*-oleoylethanolamine (OEA), which are produced through the same biosynthetic pathway as AEA and have negligible or only weak affinity for both the CB1 and the CB2 receptors, but can indirectly modulate CBR activity by interfering with endocannabinoid metabolism [45,46]. The analytical method, which was based on positive MRM and made use of the stable isotope-labelled internal standards of all analytes, provided very good lower limits of quantification (LLOQ) [41], i.e., 0.020, 0.078, 0.078, 0.195, and 0.049 pmol/mL for AEA, 2-AG, 1-AG, PEA, and OEA, respectively. Concentration values in healthy, normal-weight females (F, *n* = 76) and males (M, *n* = 45) ranged in the following intervals: AEA, 0.40–1.65 pmol/mL (F) and 0.41–1.88 pmol/mL (M); 2-AG, 0.69–3.44 pmol/mL (F) and 0.71–5.40 pmol/mL (M); 1-AG, 0.21–1.09 pmol/mL (F) and 0.26–1.78 pmol/mL (M); PEA, 7.28–28.8 pmol/mL (F) and 8.01–21.0 pmol/mL (M); and OEA, 2.41–10.20 pmol/mL (F) and 2.41–9.06 pmol/mL (M) [34]. A simpler sample preparation based on off-line SPE was proposed by Gachet et al. In addition to the aforementioned AEA, 2-AG, PEA, and OEA, they assayed for additional NAEs in human plasma, including *N*-stearoylethanolamine (SEA) and *N*-linoleoylethanolamine (LEA), prostanoids, and steroids, as well as arachidonic acid, which is functionally interlinked with different lipid signaling networks [47]. Thus, the sample extraction was based on C18 Sep-Pak SPE cartridges from Waters (Milford, MA, USA), and the following tandem mass spectrometry quantification was carried out in both the positive and the negative MRM, depending on the chemical structures of the analytes. The method was less sensitive compared to that of Fanelli et al. as the LLOQ were 0.288, 8.465, 10.702, and 0.712 pmol/mL for AEA, 2-AG, PEA, and OEA, respectively (1-AG was not taken into consideration). Nevertheless, the sensitivity was good enough to measure endogenous concentrations in plasma samples from healthy volunteers, which were 1.7 ± 0.4, 16.5 ± 5.3, 23 ± 5.2, and 9.9 ± 2.4 pmol/mL (mean concentrations) for AEA, 2-AG, PEA, and OEA, respectively. Using liquid-liquid extraction combined with UHPLC coupled to tandem mass spectrometry, Ney et al. quantified AEA, 2-AG, and OEA, along with cortisol and progesterone, in plasma samples from 121 healthy subjects, 48 females and 73 males [48]. The mean concentrations ±SD detected for AEA, 2-AG, and OEA were 0.26 ± 0.17, 14.23 ± 21.40, and 1.38 ± 0.60 pmol/mL, respectively, with males displaying higher AEA and 2AG than females in the plasma. The LLE was carried out with a 50:50 ethyl acetate:cyclohexane mixture after protein precipitation. As with Gachet et al., this assay also did not monitor the possible isomerization of 2-AG to 1-AG, which often occurs when organic solvents are used. The LLOQ were 0.069, 4.921, and 0.043 pmol/mL for AEA, 2-AG, and OEA, respectively, while the mean concentrations ±SD detected in real samples from 121 healthy subjects, 48 females and 73 males, were 0.26 ± 0.17, 14.23 ± 21.40, and 1.38 ± 0.60 pmol/mL, respectively, with males displaying higher AEA and 2AG than females in the plasma. Although no reliable plasma reference ranges are available in the literature, these results are comparable with those found by Fanelli et al. and Gachet et al., as well as other groups. Ney et al. assayed the same analytes in saliva samples from 90 healthy subjects, 55 females and 35 males, by using a method strictly derived from that used for plasma, except for the LLE, which made use of a freezer-cold 50:50 ethyl methanol:acetone mixture [48]. The LLOQ were 0.012 (LOD 0.006), 0.741, and 0.006 pmol/mL for AEA, 2-AG, and OEA, respectively, while the mean concentrations ± SD detected in real samples from 90 healthy subjects, 55 females and 35 males, were, respectively, 0.01 ± 0.01 approximately, 1.80 ± 3.07, and 0.41 ± 0.34 pmol/mL. The same research group also quantified the same compounds in hair, using liquid-liquid extraction combined with UHPLC-MS/MS [49]. Two extraction processes were needed: extraction from the hair samples and selective LLE of the analytes of interest from the hair extract. The former extraction was performed overnight with methanol, after a hair sample washing with an iso-propanol:hexane mixture, while the latter was carried in two consecutive extraction steps, with acetonitrile and chloroform, respectively. The procedure was very efficient for the ECs and NAE and good enough for cortisol and progesterone. The method offered the LLOQ of 1.15, 7.01, and 1.42 pmol/mL for AEA, 2-AG, and OEA, respectively, while the mean concentrations ± SD detected in real samples from females (n. 25) and males (n. 3) were 2.19 ± 2.16, 24.55 ± 8.60, and 353.76 ± 546.85 fmol/mg for AEA, 2-AG, and OEA, respectively.

Several methods for tissue ECs extraction have been described in the literature. The most widely used are based on those described for lipids by Folch [50] and by Bligh and Dyer [51], which have been adapted to different tissues for the assessment of ECs with mass spectrometry [27,52]. In 2019, Ita and Kelly described the characterization of cerebral cortical endocannabinoid levels in rats using liquid chromatography–tandem mass spectrometry (LC–MS/MS) [53]. Their results indicated that the physiological concentrations for AEA, 2-AG, PEA, and OEA were 0.03–0.04, 1.54–1.91, 0.46–0.56, and 0.19–0.22 nmol/g, respectively. The sample preparation included homogenization in acetonitrile in an ultrasonic bath after the addition of known fixed amounts of the stable isotope-labeled internal standards of AEA, 2-AG, PEA, and OEA, centrifugation, evaporation to dryness of the supernatant, and resuspension in 65% acetonitrile prior to the injection into the HPLC-MS/MS system. The method LLOQ were 1.32, 12.1, 1.5, and 1.41 pmol/g for AEA, 2-AG, PEA, and OEA, respectively, and the indicative ranges of the physiological concentrations deduced from the bar graphs in the paper were, respectively, 0.03–0.04, 1.54–1.91, 0.46–0.56, 0.19–0.22 nmol/g. In summary, the availability of highly sensitive analytical tools allowed a reliable determination of ECs in plasma and tissue samples and can provide a fundamental contribution to the understanding of the physiological and pathophysiological role of the ECS and its potential in the treatment of a large variety of disorders, including neurodegenerative, cardiovascular, metabolic, and bone diseases.

## 4. The Involvement of the ECS in Bone Metabolism

Our knowledge of the ECS is relatively young and the awareness of a possible link with skeletal health was initially derived from some casual observations. The first such link was correlated to leptin, the adipocyte-produced hormone, which not only centrally controls energy expenditure but also appetite. Indeed, the crosstalk between leptin and ECs in mediating the food intake has been reported, demonstrating that hypothalamic ECs are under negative control by leptin and may be considered to belong to the growing family of orexigenic (appetite-stimulating) mediators. The higher levels of ECs in genetically obese (ob/ob) and diabetic (db/db) mice support this observation. [54]. Moreover, recent evidence points to a role of leptin as an osteogenic molecule with direct effects on bone, through its receptors [55]. The idea that 2-AG, one of the two principal ECs, is produced under leptin control, led to the first connection between the two systems [55]. Moreover, 2-AG has been proposed as having a neuroprotective role after brain injury, which is also a potent stimulus for bone formation [56,57]. Indeed, in patients with brain trauma, increased osteogenesis with heterotopic ossification has been observed [58].

**Figure 3:**
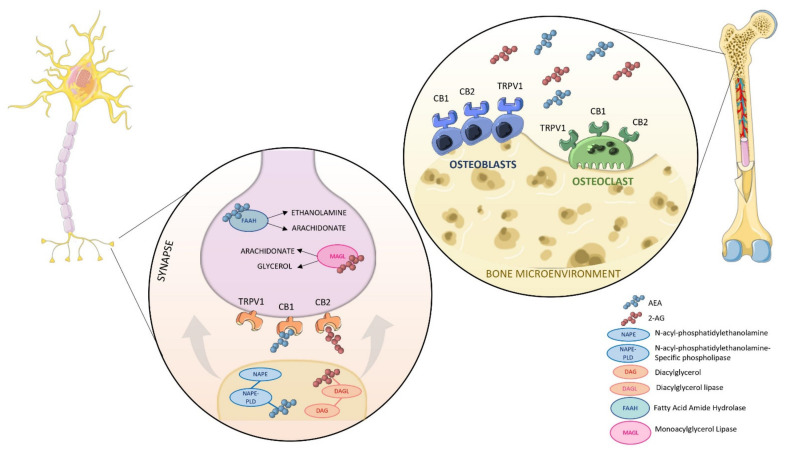
A schematic overview of the skeletal ECS. Right panel shows endocannabinoids anandamide (AEA) and 2-arachidonylglycerol (2-AG), which are found in the bone microenvironment. Endocannabinoid receptors CB1, CB2, and TRPV1 are located on osteoblast and osteoclast cell membranes. Left panel shows the sympathetic innervation of bone. Axons from dorsal root ganglion neurons travel to periosteum and cortical bone. Postganglionic synaptic structures synthesize endocannabinoids on demand and release these lipophilic compounds into the synaptic cleft, where they travel in a retrograde direction to bind to membrane receptors found on osteoblasts and osteoclasts. Osteoblasts and osteoclasts contain enzymes for endocannabinoid synthesis (NAPE, NAPE-PLD, and DAGL) and degradation (FAAH and MAGL). AEA is degraded by FAAH into arachidonate and ethanolamine, whereas 2-AG is metabolized by MAGL into arachidonate and diacylglycerol (DAG).

Apart from these links, more solid and direct evidence has been collected in the last ten years, and it is now clear that bone cells display not only CBRs, but also the entire enzymatic machinery for EC biosynthesis and can modulate local EC production and secretion according to the maturation and differentiation stage [59] (Figure 3).

**Figure 4:**
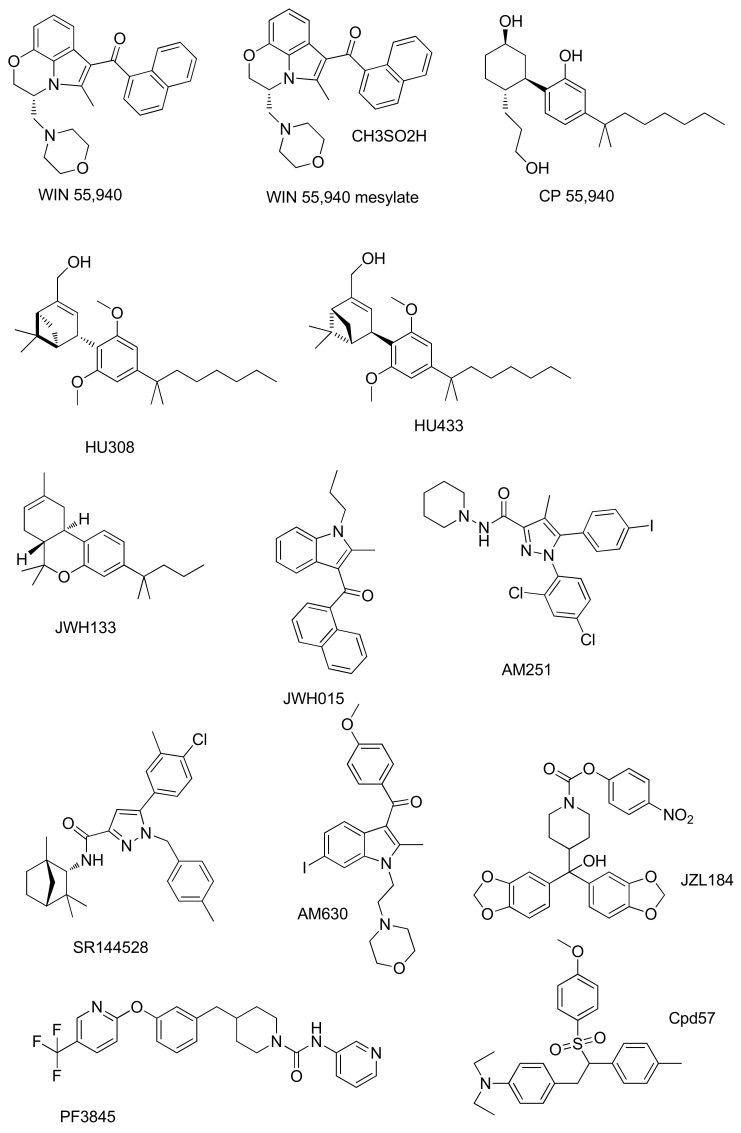
Structures of ECS synthetic modulators studied in bone diseases.

Since the relevant role of CBRs in bone metabolism was discovered, many synthetic modulators of the ECS have been developed and studied on bone diseases (Figure 4). These compounds can either activate the receptor (agonists) or inactivate it (antagonists or inverse agonists) or act on specific enzymes of the ECS, modulating indirectly the CBRs through the levels of the ECs (Table 1).

### 4.1. In Vitro Evidence of a Possible Modulation of ECS in Bone

The levels of the main ECs measured directly in the trabecular bone tissue (mouse) are in the order of 35.8 ± 6.2 pmol/g for anandamide (AEA) and 1.4 ± 0.1 nmol/g for 2-AG, as reported by Bab et al. [75]. These concentrations are similar to those measured in mouse brains but much higher than the concentrations found in the blood, suggesting a local production of both ECs in the bone [75]. In vitro AEA and 2-AG have been shown to be secreted by OBs and OCs and CB1R, CB2R, and TRPV1 have been demonstrated to be expressed by bone cells (OBs, OCs, and osteocytes) [76,77]. Rossi et al. investigated the presence and the modulation of CB2R and TRPV1 in human OBs cultures obtained from healthy donors. In these cells, they could demonstrate the presence of mature mRNA for CB1R, CB2R, and TRPV1 and for crucial enzymes in the synthesis (NAPE-PLD- and DAGL-α) and degradation of AEA and 2-AG (FAAH- and MAGL), respectively. Subsequently, they showed that the stimulation of CB2R and TRPV1 by two synthetic agonists was able to modulate osteogenic markers produced by OBs in different ways. RTX, a selective agonist of TRVP1, decreased bone matrix deposition, reducing RUNX, ALP, OPG mRNA, and proteins and calcium deposits, whereas JWH-133, a selective CB2R agonist, stimulated all these bone apposition markers. The CB2R and TRVP1 selective agonists also showed an effect on the production of RANKL by OBs, the crucial activator of OC proliferation and action. The effects of the two agonists were similar in this case and markedly decreased RANKL, suggesting a further effect on the inhibition of osteoclastogenesis and maturation. The hypothesis is that CB2R activation may act consistently on OBs (directly) and on OCs (indirectly by RANKL) as a potent inductor of bone mineralization, while TRVP1 activation may act alternatively on enhancing or diminishing bone apposition as a flexible modulator system [78]. In another cell model of OBs, namely the MC3T3-E1 osteoblast-like cell line (subclone 4), Hutchins et al. demonstrated that NAPE-PLD, FAAH, and CB2R mRNA expression increased during OB maturation, being higher at 20 days compared to 10 days. Moreover, the administration to cell cultures of eicosapentanoeic acid (EPA), which reduces the arachidonic acid (AA) precursor of AEA, actually inhibited the enzyme expression of the ECS and in turn the AEA synthesis [79]. More recently, Kostrzewa et al. used the same model MC3T3-E1 osteoblasts to evaluate the different expression of the ECs and enzymes during different phases of OB differentiation. Assuming an “on demand” EC production that depends on differential requirements during maturation [80], they demonstrated that 2-AG has a peak in pre-osteoblasts at the beginning of the differentiation period (day 0) and declines during the entire process; on the other hand, AEA is stable and detectable until day 14 and then dramatically increases with a peak in mature OBs (day 21). These findings suggest a different role of the ECS components in different moments of the OBs’ life [80].

The ECS’ presence and activity have also been demonstrated in vitro in OC cultures, primarily in murine cells [81] and subsequently in human cells. Whyte at al. evaluated the expression of CB1R and CB2R by qPCR and Western blotting in human OCs derived from monocytes enriched with M-CSF and RANK-L from healthy donors. Both CB1R and CB2R were present in monocytes and mature OCs, with CB1R being the former, constantly expressed during the differentiation process, while CB2R decreased at the end of the OC maturation. Moreover, immunocytochemistry confirmed the presence of the CBRs in OCs in the cells and at the cells’ surface, according with the receptors‘ localization [82]. AEA and 2-AG basal production was detected in mature OCs by LC-MS-MS, suggesting that the presence of both the active molecules and the receptors may have an autocrine effect on OCs. Indeed, the addition to the culture medium of AEA and 2-AG markedly decreased (up to 36 and 42%, respectively) the monocyte differentiation in mature OCs, without affecting the cells’ viability. On the other hand, AEA directly added to mature OCs was able to stimulate OC polarization and resorption, and this effect was inhibited by the administration of the CB1R and CB2R antagonists [64]. In summary, a dynamic regulation of ECS has been demonstrated in OCs in vitro, either during OC maturation or during activation of mature OCs to bone resorption.

Taken together these data were the first in vitro evidence of a possible modulation of the ECS in bone by modifying OB and OC differentiation and actions.

### 4.2. Animal Studies Provide Further Insight in the Role of CBRs in Bone Biology

Further insight into the role of CBRs has been provided by animal models of knockout mice for CB1R and CB2R, even though the experimental data are still not fully explained, and the skeletal phenotypes are dependent on strain, age, and sex.

The initial studies were performed by Idris et al., who demonstrated that young CB1R knockout (KO) mice on a CD background showed higher bone mineral density (BMD) compared with WT animals, both at the spine (+10%) and the femur (+18%), measured by dual X-ray absorptiometry (DXA) and confirmed by peripheral quantitative computed tomography (pQCT). Moreover, CB1R KO mice did not display the 40% decrease in trabecular bone volume, which was observed in WT littermates after ovariectomy, and these effects were correlated with osteoclast apoptosis and/or insufficiency [70]. The same group subsequently showed that the CB1R KO developed a severe age-related osteoporosis at 12 months, characterized by a replacement of bone marrow by adipocytes and a defect in the capacity of pre-osteoblasts to differentiate in the mature OBs. They hypothesized that in the CB1R-deficient mice a defect in the OC resorption happens during young age, while a defect of OB bone mineralization is compensated by other factors at this stage. On the other hand, in aged mice, the compensatory factors fail, and the OB differentiation is lost, resulting in reduced bone formation and osteoporosis [63]. These findings confirm that the involvement of CB1R in bone turnover is complex and probably age-related but crucial for normal skeletal phenotype. However, it should be argued that Tam et al. found a different skeletal phenotype in CB1R−/− on a different C57BL/6J background with low bone mass: the reasons for the discrepancies could be related to the fact that this strain generally displays a lower femoral volumetric bone compared to the other strains, or it could be a strain-related epigenetic effect [83].

The mechanism of the CB1R action on bone still needs to be elucidated. However, CB1R density on bone cells seems to be lower compared to CB2R distribution, while there is an abundance of CB1R on skeletal sympathetic nerve endings, which richly innervate bone in proximity to OBs. Sympathetic fibers normally suppress bone formation upon activation by norepinephrine, and CB1R probably elicits an indirect effect, reducing norepinephrine inhibition on OBs [84]. This is in line with the same EC sympathetic nerve inhibition in other organs and could open interesting scenarios in the modulation of brain-associated bone loss, such as in depression, brain injury, or anorexia [76,85].

CB2R is more densely expressed on bone cells, and the CB2R KO offers insights into related human diseases, even though there are important differences that are age- and strain-related. In 2006, Ofek et al. proposed a CB2R−/− on a C57BL/6J background model, which was healthy and fertile, but showed a low bone mass both in males and females, due to a progressive trabecular bone resorption. Indeed, bone turnover was found to be increased, with a higher OC number and activity (more than 40%) compared with a modest increase in bone formation (20%) [65]. This phenotype resembles that of post-menopausal osteoporosis in which bone remodeling is skewed towards resorption. CB2R genetic deletion has different effects in different mouse strains, as confirmed by a transcriptomic study by Sophocleous et al. [86]. Indeed, CB2R−/− on a CD1 background displays a low bone turnover early in age, with a bone volume increase compared to WT, followed by a fast bone loss with age, similar to CB2R−/− C57BL/6J [87]. It is noteworthy that independently from strain, the CB2R loss appears to profoundly alter the bone-remodeling process.

Additional interesting evidence was found with the double KO CB1R−/−CB2R−/− on a CD background which, unlike the single KO, showed a high peak of bone mass at a young age but also a protection against bone loss in older age, different from the single gene KO [88].

While significant results support the idea that age and animal strain modulate the skeletal role of CBRs, additional studies are required to better understand whether the skeletal role of CBRs has a sex-bias. In this context, it has been demonstrated that the skeletal phenotype in mice with a CB1R deficiency has a gender disparity as female mice showed normal trabecular bone with a slight cortical expansion, whereas male CB1R−/− animals displayed a high bone mass (HBM) phenotype [89]. Other reports also detected gender-specific differences in the skeletal response to CB2R deficiency. For instance, the female CB2R−/− mice had a significantly higher peak bone mass in the trabecular compartment at the tibial and femoral diaphyses compared with the wild-type littermates, whereas males of the same strain were not affected by CB2R knockout [87]. Nevertheless, the relative contribution of sex and CBR signaling in bone biology still needs to be fully elucidated.

## 5. Cannabinoid Receptors as Therapeutic Target for Bone Diseases

As illustrated above, in vitro and animal models provide compelling evidence that ECs play important roles in bone formation, bone resorption, and skeletal growth, showing a great promise in the treatment of bone diseases associated with accelerated osteoclastic bone resorption, including osteoporosis and bone metastasis.

### 5.1. Osteoporosis

Osteoporosis is a bone metabolic disease, characterized by progressive impairment in bone quantity and quality, which finally results in high morbidity and mortality related to fractures. Primary osteoporosis is a major health problem in elderly patients, but secondary osteoporosis can also affect young individuals. The most common cause is post-menopausal osteoporosis in women, where estrogen deficiency enhances a rapid increase in bone resorption followed by a reduction in bone formation, with a progressive decrease in bone density and strength [90]. Moreover, in postmenopausal osteoporosis, hypovitaminosis D (25hydroxyvitamin D or 25OHD less than 20 or 30 ng/mL, according to different guidelines) has been identified as one of the most important contributors [91]. Vitamin D is a hormone whose main effect on the skeleton is due to the stimulation of calcium absorption by the gut. Vitamin D deficiency induces secondary hyperparathyroidism with osteomalacia, bone loss, impaired bone strength, and osteoporotic fragility fractures. Vitamin D treatment has been shown to significantly improve bone density indices and reduce the incidence of osteoporosis [92]. Therefore, it is highly recommended that continuous and regular treatment with vitamin D supplements be performed, especially in women and the elderly, to prevent and even improve osteoporosis. Other risk factors for osteoporosis may include poor physical activity, low calcium intake, and obesity. It is well-established that increasing physical activity positively affects bone health, while reductions in physical activity can result in bone loss [93,94,95]. Therefore, physical activity is a viable strategy for the prevention and treatment of low bone mass and may represent an attractive alternative to medication for the treatment of osteoporosis [96]. Several lines of evidence indicate that exercise may prevent body fat accumulation while increasing bone mass. The association between the bone and adipose tissue is complex and not fully understood. The latter has recently been associated with bone microarchitecture impairment due to sarcopenic muscles, low grade systemic inflammatory status, and increased bone marrow adipogenesis, which are harmful for bone [97]. Nevertheless, a better understanding of the association between adipose and bone tissue would promote the identification of new molecular therapeutic targets that will enhance osteoblastic activity and/or inhibit adipogenesis and osteoclastic activity.

Secondary osteoporosis is mainly due to corticosteroids iatrogenic administration, endocrinological diseases, such as primary hyperparathyroidism [98,99], or other inflammatory and autoimmune diseases which negatively affect bone [100]. Despite the availability of several effective therapies, osteoporosis is an extensive problem that will greatly benefit from the development of new therapeutic approaches aimed at novel targets. The ECS could be a valid candidate, considering the above mentioned implications in bone-health maintenance [101].

Even though the connection between the ECS and bone is clear, the complexity of these interactions represents a challenge that still needs to be unraveled. Indeed, the pharmacological modulation of the ECS receptors by CBR agonists and antagonists showed different results, depending on the model in which they were tested, their selectivity on CB1R or CB2R, as well as additional cell- and time-related factors. At present, none of the several modulators that have been studied has reached human experimentation.

CB2R specific ligands are very promising as they could exert a beneficial effect on bone loss, without effects on the central nervous system. Ofek et al. tested a synthetic specific CB2R agonist, namely HU-308 with a molecular weight of 414, in an animal model of osteoporosis induced by ovariectomy (C3H mice) [65]. HU-308 was daily injected intraperitoneally at a dosage of 10 mg/kg/day for 4 weeks, starting immediately after ovariectomy, to evaluate the “preventive” potential of the drug in bone-loss attenuation. The results showed a trabecular bone loss of 41% in untreated mice and a 27% reduction in the loss in treated mice. The beneficial effect of HU-308 was shown to be related to a decrease in OC activity and OC resorption together with OB-stimulated bone apposition [65]. The same group also tested a “rescue” approach with the same CB2R analogue, HU-308, administered starting 6 weeks after ovariectomy for 4–6 weeks; in this case HU-308 was also able to attenuate ovariectomy-induced bone loss [102]. The advantage of a positive modulation of peripheral CB2R is consistent with the only available human studies on patients, which showed that genetic defects of the *CB2* gene are related to osteoporosis. The *CB2* gene is located on chromosome 1p36, and it is constituted by a single coding exon (exon 2) and an upstream noncoding exon (exon 1); its analogue in mice resides on chromosome 4QD3. These regions were found to be related to BMD or osteoporosis in association studies by Devoto et al. [103,104,105]. Subsequently, in 2005 Karsak et al. evaluated 168 women with osteoporosis and 220 matched controls in whom were analyzed the difference in the distribution of 26 single nucleotide polymorphism (SNPs) distributed into the *CB2* gene and in the surrounding genomic region (300 kb around). Many of the evaluated SNPs were found to be associated with the osteoporosis phenotype. Moreover, the best statistical association was present for the two SNPs present in the coding region of the *CB2* gene, and the lumbar spine BMD was significantly lower in patients harboring these specific SNPs as compared to the others. The functional hypothesis from the authors was that at least some of the evaluated SNPs could alter the receptor function as two of the selected SNPs associated with osteoporosis were missense variants with amino acid substitutions (His316Thyr and Gln63Arg). On the other hand, in the same study the *CB1* gene was also analyzed, but no association was found between selected SNPs and the osteoporosis phenotype [106]. The same group performed another study with a family-based design, in a population of 574 adults, 277 women, and 290 men aged 18–90 years, all belonging to Chuvashians families from the same area of Russia and showing very stable genotypic characteristics and environmental conditions. They found a significant association between the two, among 16, *CB2* SNPs evaluated and the bone features risk indexes, namely the radiographic hand BMD and the breaking bending resistance index (BBRI), which are indicators of bone strength or fragility [107].

In order to assess the susceptibility to osteoporosis in patients carrying *CB2* SNPs, Yamada et al. designed a prospective study in which they evaluated 1110 women and 1128 Japanese men aged 40–79 years for the association between BMD at different sites and the presence of SNP rs2501431, which was the most promising in the Karsak cohort [108]. Rs2501431 leads to a change of A- > G, and the GG genotype was found to be associated with significantly lower BMD at the distal radius, lumbar spinal, and femoral neck compared to the AA genotype in pre- and postmenopausal women [108].

There are two more studies that confirmed the same findings: in a Chinese study from Huang et al., they studied four genes on chromosome 1p36, namely *TNFRSF1B, PLOD, CNR2 (CB2*), and *MTHFR* in a large case-control population of 1243 subjects. Among the evaluated genes, *CB2* SNP rs2501431 (A592G; G155G) were found to be associated with femoral neck BMD [109]. In a more recent study on a Korean cohort of 470 post-menopausal women, *CB2* rs2501431, rs3003336, rs2229579, and rs4237 polymorphisms showed an association with lumbar spine BMD. In particular, women harboring rs3003336 and rs4237 SNPs had significantly lower BMD compared with the others. In this study, bone turnover markers were also measured but did not show any association with the genetics of *CB2* [110].

### 5.2. Bone Cancer

**Figure 5:**
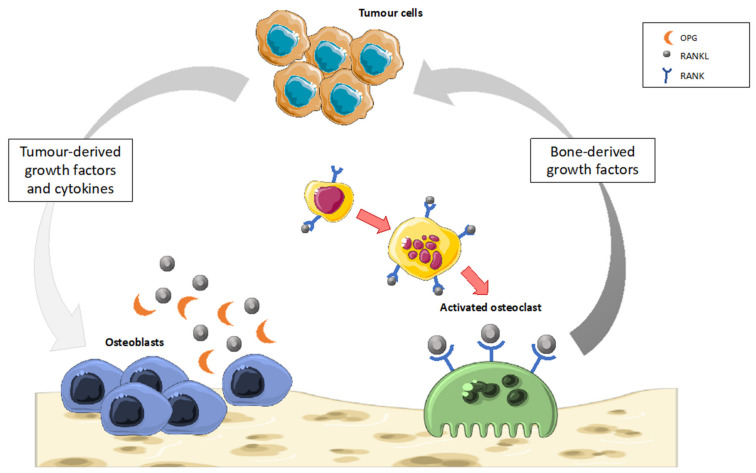
An overview of the “vicious cycle” balance in CIBD. Cancer cells release several factors that stimulate OBs to secrete RANKL. The over-expression of RANKL in OBs drives osteoclast-mediated bone resorption and the consequent release of numerous survival factors, such as insulin-like growth factor 1 (IGF-1) and transforming growth factor beta (TGF-β), which in turn promote the survival and proliferation of tumor cells, thus potentiating cancer spread and bone destruction.

Cancer-induced bone disease (CIBD) is a common and severe complication of cancer, which is burdened by high morbidity and mortality due to disabilities, bone pain, immobilization, nerve impairment, fragility fractures, and malignant hypercalcemia [111]. CIBD is generally secondary to bone metastases by primary solid tumors, such as breast or prostate cancer, which metastasize in bone in up to 80% of cases; less frequently, it is due to primary bone cancer and hematological malignancies [112]. Regardless of the phenotype of bone metastases (i.e., osteolytic vs. osteoblastic), they are the result of a severe deregulation of the bone remodeling process that results in a “vicious cycle” between bone and tumor cells [113] (Figure 5). The release of tumor-derived factors, such as parathyroid hormone-related protein (PTHrP), interleukin 6 (IL-6), tumor necrosis factor (TNF), and transforming growth factor β (TGF-β), activates OC differentiation and bone-resorbing activity. Bone resorption results in the release of bone-derived growth factors, cytokines, bone extracellular matrix components, further supporting tumor proliferation and bone destruction [114].

This mechanism increases RANKL-mediated osteoclast activity, leading to osteolytic lesions, which are typical, for instance, in breast cancer metastases [115]. In contrast, there are OB phenotypes that secrete growth factors, such as endothelin-1 (ET-1) and bone morphogenic proteins (BMPs) that selectively stimulate osteoblastic proliferation, leading to increased bone formation. This mechanism instead decreases RANKL-mediated osteoclast activity, leading to osteoblastic lesions, which are typical, for instance, in prostate cancer metastases [116]. Therefore, the cancer cells disrupt the balance between RANKL and OPG in the bone environment, leading to an excess of bone resorption and loss (osteolytic lesions) or apposition of abnormal bone (osteoblastic lesions) [117]. The RANKL level is upregulated in osteolytic lesions associated with malignant tumors, whereas the OPG level is upregulated in osteoblastic lesions [118]. Current available therapies mainly inhibit osteoclastic bone resorption due to the tumor but are not very effective in controlling bone pain. The ECS is known to be implicated in the cancer growing process, in pain regulation, and metastasis induction, and the modulation of CB2R could be a potential novel therapeutic target for CIBD [119].

Evidence has been reported that CB2R agonists have an inhibitory effect on tumor cell growth and lead to tumor cell death in vivo and in vitro: the mechanisms responsible for these actions are not fully elucidated but have been related to the inhibition of vascular growth factor (VEGF) produced by tumor cells, reduction in the proteolytic matrix metalloproteinases MMP1, MMP2, and MMP9, and inhibition of angiopoietin 2 [120,121]. The selective agonists of CB2R, namely JWH-133, have been demonstrated to reduce tumor growth and invasion in different osteosarcoma cell line models, alone or as a beneficial co-adjuvant of bortezomid, a potent proteasome inhibitor drug [69,122]. Moreover, JWH-133, as well as HU308, have been demonstrated to decrease osteotropic breast cancer cell proliferation (4T1 mice cell line and MDA-MB-231 human cell line) in a dose-dependent manner [67]. The selective agonist JWH015 demonstrated a very interesting anti-proliferative effect as it significantly reduced the number of breast cancer cell line 66.1 after injection into the intramedullary cavity of mice to mimic bone metastasis [123]. Anandamide itself, which is a non-selective CBR agonist, has been proven to induce apoptosis in osteosarcoma cell lines by p38 MAPK and caspase-3 activation intracellular signaling [124]. In line with a previously demonstrated activity of CBRs to activate the autophagy mechanism in vitro, another non-selective agonist for CB1R and CB2R, namely WIN55,212–2, showed a beneficial effect in inducing osteosarcoma cell death by the autophagy mechanism and was a potent adjuvant of other anticancer drugs, such as Adriamycin [61,125].

Modulation of CB2R also seems to play a role in the regulation of bone-cancer-induced osteolysis. Indeed, JWH015 was tested in a mouse model of breast cancer in which it was able to reduce bone metastases, fracture from bone metastasis, and markers of bone turnover. AM1241 had similar beneficial effects on bone osteolytic damage and fracture in a mouse model of sarcoma. Unfortunately, to date the mechanism for the action of these CB2R modulators on bone is not fully understood as it is also not clear whether they act as partial/full agonist or antagonists of CB2R [111,123,126].

## 6. Conclusions

Considering the involvement of the ECS in bone remodeling, pharmacological modulation of the ECS could offer a possible treatment for pathological conditions where an altered OB/OC activity is observed. A large variety of ECS synthetic modulators is currently available. Among them, CB2R specific ligands are very promising for their beneficial action on bone loss, without causing adverse psychotropic effects. However, before large-scale human studies can be conducted, further investigations in animals are needed to delineate their pharmacokinetic properties, as well as the safety and toxicity profiles.

In human studies, CB2R activation has been shown to protect from osteoporosis. In addition, the polymorphism in the *CB2* gene has been proposed as a diagnostic tool to detect genetic predisposition to osteoporosis in humans. Moreover, increasing evidence suggest that agents that target CB2R in the skeleton have the potential to reduce the skeletal complications associated with cancer. Therefore, the development and testing of CB2R selective ligands in preclinical models of metastatic cancer will pave the way for future research that will advance our understanding about the mechanism(s) by which the ECS regulates cancer metastasis and offer novel therapeutic options to reduce skeletal tumor burden.

## Figures and Tables

**Table 1:** Synthetic modulators of the ECS and their effects on bone metabolism.

Compound	Activity	Experimental Model	Effect on Bone	References
**WIN55,212**	CB1R agonism	Murine MC3T3-E1 osteoblasts.	Glucocorticoid-induced inhibition of mineralization in MC3T3-E1 osteoblasts	[60]
	CBR agonism	MG-63 human osteosarcoma cell line.	Antitumor activity and its combined effect with adriamycin against the MG-63 human osteosarcoma cell line	[61]
**WIN55,212-2 mesylate**	CBR agonism	Human chondrocytes.	Prevention of cartilage breakdown in arthritis	[62]
**CP55940**	CB1R agonism	Bone marrow mouse cell.	Stimulation of bone nodule formation	[63]
	CB2R agonism	Human osteoclasts.	Inhibition of human and mouse osteoclast formation Stimulation of human osteoclast polarization and resorption	[64]
**HU308**	CB2R agonism	Bone-marrow-derived osteoblasts/stromal cells. Osteoblasts isolated from mouse calvarial bones	Increased osteoblast differentiation and activity	[65,66]
		OVX C3H mouse model of postmenopausal osteoporosis.	Prevention of osteoporosis in ovariectomized rats	[14,65,66]
		M-CSF-generated osteoclasts	At low concentration stimulation of osteoclast formation. At high concentrations of inhibition of osteoclast formation	[14]
		4T1 mice cell line and MDA-MB-231 human cell line	Reduction in osteotropic breast cancer cells proliferation	[67]
**HU433**	CB2R agonism	Newborn mouse calvarial osteoblasts	Stimulation of osteoblast proliferation	[68]
		Osteoclastogenic cultures from bone marrow-derived monocytes	Increase in osteoclast apoptosis	
		Ovariectomized-mouse model	Prevention of osteoporosis in ovariectomized mouse	
**JWH 133**	CB2R agonism	M-CSF-generated osteoclasts	Stimulation of osteoclast formation	[14]
		Six different OS cell lines	Anti-proliferative, pro-apoptotic, anti-invasive effect.	[69]
		4T1 mice cell line and MDA-MB-231 human cell line	Reduction in osteotropic breast cancer cells proliferation	[67]
**JWH015**	CB2R agonism	Human osteoclasts	Stimulation of human osteoclast polarization and resorption	[64]
**AM251**	CB1R antagonism	Mouse osteoclast cultures	Inhibition of osteoclast formation	[70]
		Ovariectomized-mouse model	Protection against ovariectomy induced bone loss	
**SR144528**	CB2R antagonism	Mouse osteoclast cultures	Inhibition of osteoclast formation	[70]
		Ovariectomized-mouse model	Protection against ovariectomy induced bone loss	
**AM630**	CB2R antagonism	Mouse osteoclast cultures	Inhibition of osteoclast formation	[70]
**Cpd 57**	CB2R inverse agonism	Osteoclast from RAW 264.7 cells	Inhibition of osteoclast formation	[71]
**JZL184**	MAGL inhibition	Osteoclast from RAW 264.7 cells	At low concentration stimulates osteoclast formation. At high concentrations inhibits osteoclast area	[72]
		Human osteoblast-like cells Saos-2	Inhibition of osteoblastic bone formation No effect to mature or form bone nodules	
		Bone marrow macrophages (BMMs)	Suppression of osteoclast differentiation and function	[73]
		C57BL/6 mice	Reduction OVX-Induced Bone Loss	
**PF-3845**	FAAH inhibition	Osteoclast from mouse bone marrow macrophages	Suppression of osteoclast differentiation. Anti-resorptive activity prevents alveolar bone loss	[74]
		male C57BL/6 mice	Prevention alveolar bone loss	

## Data Availability

Not applicable.
